# Pentachlorophenol, polychlorinated dibenzo-p-dioxins and polychlorinated dibenzo furans in surface soil surrounding pentachlorophenol-treated utility poles on the Kenai National Wildlife Refuge, Alaska USA

**DOI:** 10.1007/s11356-018-2269-7

**Published:** 2018-06-01

**Authors:** Lori A. Verbrugge, Lynnda Kahn, John M. Morton

**Affiliations:** 1 U.S. Fish and Wildlife Service, Alaska Regional Office, 1011 E. Tudor Rd, Anchorage, AK USA; 2 U.S. Fish and Wildlife Service, Kenai National Wildlife Refuge, P.O. Box 2139, Soldotna, AK USA

**Keywords:** Pentachlorophenol, Polychlorinated dibenzo-*p*-dioxins, Polychlorinated dibenzo furans, Utility pole, Alaska, Soil

## Abstract

Composite surface soil samples were collected at 0, 25, and 50 cm from the base of 12 utility poles on the Kenai National Wildlife Refuge in Alaska, to assess the extent to which pentachlorophenol, polychlorinated dibenzo-*p*-dioxins and polychlorinated dibenzo furans may have leached from pentachlorophenol-treated poles. Six pairs of utility poles were included, consisting of an “old” pole manufactured in 1959 or 1963, a “new” pole manufactured within the past 20 years, and a suitable background soil sample from the same vicinity. Old poles had greater concentrations of 2,3,7,8-tetrachlorodibenzo-*p*-dioxin (TCDD) equivalents (TEQs) near the pole base and at 25 cm than “new” poles did. For all 12 poles combined, the mean pentachlorophenol levels in soil were 1810, 157, and 17.8 ppm dry weight (d.w.) near the pole bases, at 25 and 50 cm from the poles, respectively, while the mean total TEQ levels in soil were 15,200, 5170, and 1510 parts per trillion d.w. at those distances. Surface soil levels of pentachlorophenol and TCDD-TEQs exceeded both human health and ecological risk-based screening levels. The design and results of this study were similar to another project in Montreal, Quebec in Canada. Together the results are cause for concern, indicating that millions of similarly treated utility poles in North America may be point sources of pentachlorophenol and dioxins/furans to soil.

## Introduction

In North America, pentachlorophenol has been used as a wood preservative since 1936. While pentachlorophenol is a general biocide that has been used for a variety of purposes in the past, its only remaining use in the USA is as a heavy-duty wood preservative, particularly for wood utility poles and cross arms (USEPA 2008). In 1992, there were estimated to be 36 million pentachlorophenol-treated utility poles in service in the USA (Malecki 1992). Commercial pentachlorophenol mixtures used to treat wood are known to contain polychlorinated dibenzo-*p*-dioxins and polychlorinated dibenzo furans (PCDDs and PCDFs); the concentration of these contaminants has decreased since pentachlorophenol became more strictly regulated by the US Environmental Protection Agency (USEPA) in 1987. In 1987, the USEPA established that commercial pentachlorophenol products in the USA could not contain more than 4 ppm of hexachloro-dibenzo-*p*-dioxins (HxCDD) or exceed 2 ppm HxCDD as a monthly average (Eduljee 1999). Pentachlorophenol-treated utility poles can contain substantial quantities of dioxins and furans and are an important reservoir source of these toxic chemicals with the potential to contaminate the environment (Lorber et al. 2002).

PCDDs and PCDFs are a class of structurally similar compounds that are toxic to a wide variety of organisms. Laboratory animals experimentally exposed to PCDDs and PCDFs have exhibited dermal, immunological, and hepatic toxicity; teratogenic, carcinogenic, and neurobehavioral effects; endocrine disruption; and biochemical changes including induction of several drug-metabolizing enzymes (Ahlborg et al. 1992). They share a common mechanism of toxicity, and the relative toxicity of each congener is based on its structural ability to bind with the Ah receptor, which mediates toxicity (Safe 1990). Toxic equivalency factors (TEFs) have been developed for each congener, which express each congener’s toxicity relative to 2,3,7,8-tetrachlorodibenzo-*p*-dioxin (TCDD), the most potent PCDD (Van den Berg et al. 1998, 2006). The overall toxicity of a complex mixture of PCDDs and PCDFs can be calculated from measured congener concentrations and expressed as TCDD-equivalents or TCDD-TEQs. PCDDs and PCDDs are environmentally persistent and lipophilic and biomagnify in aquatic food chains (Ahlborg et al. 1992).

Pentachlorophenol uncouples oxidative phosphorylation, which interferes with cell respiration and results in a marked increase in metabolism (Holmberg et al. 1972; Eisler 1989). Oxygen radicals play a central role in the generation of lipid peroxidation; in rats the primary metabolite of pentachlorophenol (tetrachlorohydroquinone) is more toxic than the parent compound (Wang et al. 2001). Pentachlorophenol was classified by the US EPA as “likely to be carcinogenic to humans” during its last status review in 2010 (USEPA 2010). Pentachlorophenol exhibits endocrine-disrupting effects at environmentally relevant concentrations, including anti-estrogenic and anti-androgenic activities at low exposure concentrations in vitro and decreased ovulation in vivo (Orton et al. 2009). Human exposure to pentachlorophenol decreased significantly in North America following regulatory restrictions. Pentachlorophenol levels in blood from North Americans had a geometric mean of 123.26 μg/L in the 1980s which fell to a geometric mean of 1.36 μg/L after 1995 (Zheng et al. 2011).

Pentachlorophenol accumulates rapidly in exposed fish, with uptake primarily from water rather than from the diet (Niimi and Cho 1983). Environmental exposures to high levels of pentachlorophenol have resulted in fish kills, bird deaths, and poisoning of livestock (Eisler 1989). At lower concentrations more typically found in the environment, pentachlorophenol may have adverse effects on the reproductive and inter-renal systems of exposed fish. Fish exposed to environmentally relevant concentrations of pentachlorophenol for 28 days showed changes in steroid hormone levels in plasma, inhibition of spermatogenesis in male fish, and degeneration of ovaries in female fish (Yang et al. 2017). Species sensitivity distributions provide helpful information about the relative toxicity of pentachlorophenol to various aquatic species (Jin et al. 2012).

Given the toxicity of commercial pentachlorophenol mixtures, the environmental fate of pentachlorophenol, PCDDs and PCDFs from in-service utility poles is of interest. Several studies have documented that pentachlorophenol (EPRI 1995), PCDDs, and PCDFs (Gurprasad et al. 1995; Bulle et al. 2010) migrate from treated poles into nearby soils. Wood treated with pentachlorophenol may release the compound through volatilization or leaching. Leaching can occur as pentachlorophenol moves down the outside of the pole along with rainwater or pentachlorophenol can move with its carrier solvent with the downward force of gravity, either at the surface or within the pole (USEPA 2008). In water systems, pentachlorophenol does not undergo hydrolysis in water at pH 4 to 9 (USEPA 2008), but it does rapidly photo-degrade in the presence of direct sunlight (Choudhury et al. 1986). PCDDs and PCDFs are environmentally persistent, with estimated soil half-lives ranging from 17 to over 100 years depending on the congener and estimated water half-lives ranging from 166 days to 21 years (Sinkkonen and Paasivirta 2000).

The 800,000-ha Kenai National Wildlife Refuge (KENWR) is located on the Kenai Peninsula in southcentral Alaska, USA (60° N, 150° W). Mountains and glaciers characterize the eastern and southeastern portions of the Refuge. The Kenai Lowlands, on the western portion of the Refuge, are primarily permafrost-free beneath a cap by silt loam derived from post-glacial windblown loess (USFWS 2010). The Lowlands consist of wetlands and mixed boreal forest dominated by black spruce (*Picea mariana*), white spruce (*Picea glauca*), white birch (*Betula neoalaskana*), and quaking aspen (*Populus tremuloides*). The climate is boreal with a maritime influence. Temperatures are rarely greater than 26 °C in summer or less than − 18 °C in winter. The frost-free growing season varies from 71 to 129 days depending on location, with about 480 mm of total precipitation per year (USFWS 2010). Abundant wildlife occur in the Refuge, including moose, bears, mountain goats, Dall sheep, wolves and other furbearers, salmonids and other fish, and other migratory and non-migratory birds.

The Kenai Lowlands are bisected by the Sterling Highway that was constructed during 1947–1951. Along most of the highway segment that runs east to west through KENWR lies the utility corridor that provides electricity to communities on the western peninsula. A local member-owned utility company has operated electric utility corridors within the KENWR under US Fish and Wildlife Service-issued Right-of-Way (ROW) Permits for many decades. Much of the ROW within the Refuge occurs in wetlands that serve many ecological functions, including spawning and rearing habitat for juvenile salmonids. Most of the utility poles in the Refuge ROW were treated with pentachlorophenol prior to being placed into service.

We undertook this study to determine whether pentachlorophenol, PCDDs and PCDFs have leached from the poles into adjacent soils on the KENWR, and if so to what extent. Refuge managers need this information to make decisions about poles that are being decommissioned, replacement poles being installed, and potential risks to humans and wildlife from contaminated soils on KENWR. We aimed to address two research questions: (1) How far have pentachlorophenol, PCDDs, and PCDFs migrated from poles at the soil surface and at what concentrations are they found? (2) Is there a difference in surface soil contaminant concentrations next to poles installed in the 1950s, relative to poles installed within the past 20 years?

## Methods

Surface soil sampling was conducted in the KENWR ROW. Our experimental design consisted of six sets of poles, of two poles each. For each set, we identified a location where a pentachlorophenol-treated pole manufactured in 1959 or 1963 was in close proximity to a pentachlorophenol-treated pole installed less than 20 years ago. We collected a background soil sample for each set of poles, located between the two poles and with qualitatively similar moisture content, vegetative cover, and soil type. In order to qualify for study inclusion, poles had to be within the KENWR boundary and could not be submerged under water, and the preservative treatment type and year had to be confirmed by reading the manufacturer “button” embedded in the pole. Since the utility corridor crosses vast seasonal wetland areas, the requirement to have dry sampling locations was a slight challenge. We attempted to identify promising pole candidates using the utility’s records, but on-the-ground surveillance was essential to the selection of poles meeting the study criteria. All samples were collected the week of 15 June 2015.

**Fig. 1:**
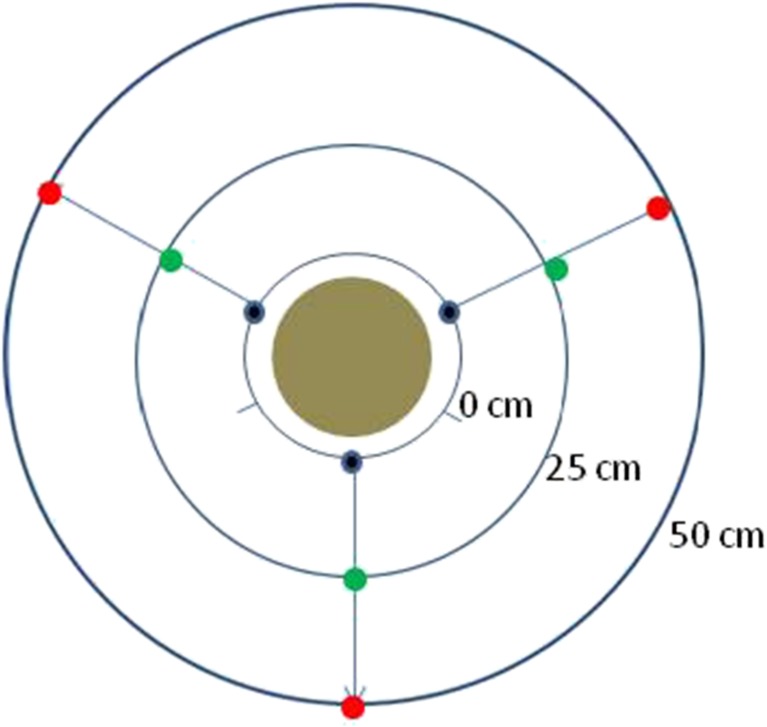
Surface soil sample points located around pentachlorophenol-treated utility poles. The three samples collected at each distance (at 0°, 120°, and 240°) were composited; hence, there was one composite sample collected at 0 cm, at 25 cm and 50 cm from each pole

Our sampling design was similar to that of Bulle et al. (2010), except we only sampled at the soil surface and not at depth. Soil samples were collected around each pole following three axes: 0° (magnetic north), 120°, and 240°, at three distances from the pole: next to the pole (at a distance between 0 and 5 cm) at 25 cm and 50 cm (Fig. 1). At each distance from the pole, the samples from the three compass points were mixed together to form a composite sample. Extra soil was collected at the 25-cm distance from two of the poles and submitted to each of the two laboratories as blind duplicates using unique sample identification numbers. Following careful removal of pebbles, vegetation, and roots and thorough mixing in a stainless steel bowl, an aliquot of each sample (approximately 100 g) was placed in each of two chemically clean amber glass bottles. Samples were placed in a chilled cooler during the field-work day, and then transferred to a − 20 °C freezer for storage.

All sampling equipment (stainless steel bowls, spoons, heavy-duty spoons, and small trowels) was precleaned in an analytical laboratory and was not used for more than one composite sample. This obviated the need for cleaning sampling equipment while in the field and eliminated the potential for cross-sample contamination. Equipment was prepared by washing in a phosphate-free soap solution (Liquinox®), rinsing in tap water, rinsing in purified water (Barnstead Nanopure Infinity), rinsing with acetone (Burdick & Jackson ‘Purified Plus’ certified ACS Grade), rinsing with high-purity hexane (Burdick & Jackson GC^2^), and allowing to air-dry completely. Aluminum foil was likewise acetone and hexane rinsed and allowed to air dry completely. Each piece of cleaned sampling equipment was then wrapped in a piece of cleaned aluminum foil, with all sampling surfaces touching the dull side of the aluminum foil, prior to transport to the field. Sample jars were purchased as precleaned and quality assured by the manufacturer for use with semi-volatile organic analytes (straight-sided wide mouth jars, 120 mL Amber glass with Teflon®-lined solid caps, C&G Scientific Containers, VWR). Samples were frozen at − 20 °C, shipped overnight on gel ice packs to two separate analytical laboratories, and then stored at − 20 °C until analysis.

Pentachlorophenol, select polycyclic aromatic hydrocarbons, and total organic carbon (TOC) were analyzed by ALS Global—Environmental laboratory in Kelso, WA (USA). EPA Method 3541 was used to extract pentachlorophenol and polycyclic aromatic hydrocarbons from soil into 1:1 (*v*/*v*) acetone/hexane using a Soxhlet apparatus. The extracted samples were then analyzed using EPA Method 8270D. Briefly, samples were injected onto a narrow-bore fused-silica capillary column that was temperature programmed to separate the analytes and detected by a mass spectrometer. Identification of target analytes was accomplished by comparing their mass spectra with the electron impact spectra of authentic standards and quantified by comparing the response of a major quantitation ion relative to an internal standard using a 5-point calibration curve. TOC was measured using EPA Method 9060. Samples were combusted in an oxygen atmosphere to convert all organic and inorganic forms of carbon to carbon monoxide (CO). The combustion product gases are swept through a barium chromate catalyst/scrubber to ensure that all of the carbon is oxidized to CO, and other potentially interfering product gases such as SO_*x*_, HX, and NO were removed from the gas stream in a series of chemical scrubbers. CO was determined using an infrared detector.

PCDD/F congeners were analyzed by AXYS Analytical Services in Sidney, British Columbia (Canada) using EPA Method 1613B. Each sample was spiked with an aliquot of cleanup surrogate solution containing ^13^C4-2,3,7,8-TCDD and extracted in a Soxhlet apparatus using 80:20 toluene/acetone. The resulting extract was cleaned up on a series of layered chromatographic columns consisting of silver nitrate/acid/base silica and alumina/carbon/Celite®. The final extract was spiked with an aliquot of recovery standard solution containing ^13^C12-TCDDs prior to instrumental analysis. Sample extracts were analyzed using high resolution gas chromatography/high resolution mass spectrometry detection. Two masses from the molecular ion cluster were used to monitor each of the target analytes and ^13^C12-labeled surrogate standards. Five additional ions were monitored to check for interference from chlorinated diphenyl ethers. A second gas chromatograph column was used for confirmation of 2,3,7,8-TCDF identification. A 5-point calibration was used. The internal standard method was used for quantification; final analyte concentrations were recovery-corrected based on the surrogate standard recovery within each sample.

Concentrations of individual PCDD and PCDF congeners were multiplied by their TEFs for human health (Van den Berg et al. 2006) to calculate TCDD-TEQs; the total 2,3,7,8-TCDD-like potency of each sample was calculated by summing the TCDD-TEQs for each sample. Limits of detection for individual PCDDs and PCDFs were mostly at the single part-per-trillion (ppt) level and were sample specific. Less than 7% of the 714 data points for individual PCDD and PCDF congeners were below the sample-specific detection limit; non-detect values were substituted with a zero for statistical analysis. We used a two-way analysis of variance (ANOVA) with interaction term (Proc GLM, SAS 9.4) to examine TCDD-TEQ variance attributable to two class variables: Age (Old, New) and Distance (0, 25, and 50 cm from pole and background values). Relative percent differences were calculated for the pentachlorophenol and TCDD-TEQ results for the two blind duplicate soil sample pairs.

## Results

Dioxin and furan congeners and pentachlorophenol were quantified in surface soil surrounding all 12 study poles (Table 1). The ANOVA (*F* = 3.12, *df* = 7,40, *P* = 0.010) suggested that pentachlorophenol concentrations within the surface soil samples neither varied by age of the poles (*P* = 0.722) or the interaction between age and distance (*P* = 0.811) but did decrease with distance from the pole (*P* < 0.001). Mean pentachlorophenol concentrations were 1810, 157, and 17.8 ppm dry weight (d.w.) near the pole bases, at 25 cm and 50 cm from the poles, respectively (Table 2). Pentachlorophenol was only detectable in one out of six background samples, at a concentration of 0.150 ppm. Sample-specific detection limits for pentachlorophenol in the other five background samples ranged from 0.066 to 0.580 ppm..

**Table 1:** Chemical analysis results for surface soil samples located near the twelve selected poles (dry wt)

	Pole 1 (new—2006)			Pole 2 (old—1963)			BG for poles 1 and 2	Pole 3 (new—1998)			Pole 4 (old—1959)			BG for poles 3 and 4	Pole 5 (new—2009)			Pole 6 (old—1959)			BG for poles 5 and 6	Pole 7 (new—2011)			Pole 8 (old—1959)			BG for poles 7 and 8	Pole 9 (new—2011)			Pole 10 (old—1959)			BG for poles 9 and 10	Pole 11 (new—2009)			Pole 12 (old—1959)			BG for poles 11 and 12
Distance (cm)	0	25	50	0	25	50		0	25	50	0	25	50		0	25	50	0	25	50		0	25	50	0	25	50		0	25	50	0	25	50		0	25	50	0	25	50	
TOC (%)	4.80	4.60	3.60	9.10	14.6	12.7	8.23	9.43	4.06	3.66	29.8	17.6	21.2	54.4	26.3	20.4	20.8	10.3	12.3	5.27	16.9	6.12	4.63	5.94	41.8	3.27	2.3	31.5	7.39	0.98	2.12	12.4	23.3	6.66	14.3	23.4	11.7	12.8	14.6	1.91	10.8	1.59
1,2,3,4,6,7,8-HpCDD (pg/g)	2.45E+05	2.56E+04	2.25E+04	5.25E+03	2.42E+04	1.06E+04	2.32E+01	9.77E+05	4.53E+05	1.87E+05	1.17E+06	2.99E+05	2.09E+05	1.27E+03	3.70E+05	6.96E+03	2.10E+03	7.55E+05	2.70E+05	2.38E+04	5.86E+01	8.69E+04	6.02E+02	4.83E+02	6.32E+05	3.81E+04	9.41E+03	1.53E+02	2.93E+05	4.80E+04	1.52E+04	9.20E+05	7.60E+05	4.27E+04	9.75E+01	2.42E+05	2.44E+03	1.85E+02	8.67E+05	5.88E+04	6.69E+04	1.74E+01
1,2,3,4,6,7,8-HpCDF (pg/g)	1.07E+05	1.24E+04	1.08E+04	1.76E+03	7.80E+03	3.38E+03	6.93E+00	9.91E+04	7.58E+04	3.18E+04	1.57E+05	1.03E+05	7.12E+04	2.37E+02	5.70E+05	1.04E+04	2.81E+03	3.11E+05	9.90E+04	8.48E+03	1.58E+01	1.35E+05	8.25E+02	3.06E+02	2.71E+05	1.48E+04	3.62E+03	4.33E+01	4.26E+05	7.18E+03	1.22E+04	3.62E+05	3.37E+05	1.59E+04	2.35E+01	4.45E+05	8.72E+02	9.12E+01	4.11E+05	2.37E+04	2.61E+04	3.38E+00
1,2,3,4,7,8,9-HpCDF (pg/g)	1.11E+04	1.07E+03	9.20E+02	1.18E+02	5.84E+02	2.47E+02	5.17E−01	2.31E+04	1.01E+04	2.92E+03	1.38E+04	5.08E+03	3.86E+03	1.89E+01	1.92E+04	1.99E+02	9.47E+01	2.04E+04	5.61E+03	4.80E+02	7.76E−01	7.56E+03	5.36E+01	1.17E+01	1.36E+04	6.89E+02	2.39E+02	2.86E+00	1.73E+04	4.28E+02	2.87E+02	2.37E+04	1.84E+04	1.03E+03	1.21E+00	1.03E+04	6.16E+01	2.85E+00	2.27E+04	1.29E+03	1.18E+03	1.70E−01
1,2,3,4,7,8-HxCDD (pg/g)	5.49E+02	2.84E+02	2.61E+02	1.14E+02	3.08E+02	2.19E+02	3.03E−1	3.20E+03	7.15E+03	3.29E+03	2.01E+04	2.81E+03	1.94E+03	2.17E+01	1.41E+03	5.67E+01	1.26E+01	4.55E+03	2.70E+03	2.87E+02	9.48E−01	3.08E+02	4.56E+00	8.15E+00	6.60E+03	3.56E+02	9.80E+01	2.28E+00	9.05E+02	4.97E+02	1.05E+02	4.52E+03	1.03E+04	4.93E+02	1.57E+00	1.38E+03	2.59E+01	2.94E+00	8.59E+03	5.07E+02	6.59E+02	2.94E−01
1,2,3,4,7,8-HxCDF (pg/g)	3.26E+03	1.22E+03	7.65E+02	5.21E+02	2.43E+03	1.06E+03	1.16E+00	1.62E+03	3.18E+03	1.17E+03	8.57E+03	6.20E+03	4.21E+03	9.50E+00	1.48E+04	3.29E+02	6.45E+01	1.47E+04	6.78E+03	6.34E+02	1.11E+00	2.53E+03	2.73E+01	1.53E+01	1.77E+04	8.88E+02	2.62E+02	3.47E+00	9.86E+03	1.22E+03	4.91E+02	1.20E+04	2.30E+04	9.83E+02	1.77E+00	1.41E+04	3.02E+01	3.75E+00	2.49E+04	1.37E+03	1.66E+03	3.04E−01
1,2,3,6,7,8-HxCDD (pg/g)	8.11E+03	1.32E+03	1.28E+03	2.62E+02	1.09E+03	6.43E+02	1.00E+00	8.43E+03	1.46E+04	5.79E+03	2.41E+04	9.71E+03	7.22E+03	4.20E+01	1.12E+04	2.24E+02	6.10E+01	2.32E+04	9.76E+03	7.60E+02	2.24E+00	1.72E+03	1.64E+01	1.97E+01	2.41E+04	1.12E+03	3.67E+02	5.96E+00	7.82E+03	2.06E+03	4.86E+02	2.15E+04	3.51E+04	1.63E+03	3.90E+00	7.49E+03	6.64E+01	6.59E+00	3.40E+04	1.75E+03	2.13E+03	7.12E−01
1,2,3,6,7,8-HxCDF (pg/g)	6.47E+02	3.56E+02	2.84E+02	1.42E+02	5.20E+02	2.32E+02	3.04E−1	5.49E+02	2.11E+03	1.03E+03	3.35E+03	2.05E+03	1.39E+03	8.44E+00	1.76E+04	5.10E+02	7.68E+01	4.70E+03	2.39E+03	2.23E+02	5.12E−01	2.82E+03	3.36E+01	1.77E+01	6.46E+03	3.28E+02	8.84E+01	1.75E+00	1.03E+04	4.87E+02	4.10E+02	4.22E+03	9.53E+03	3.83E+02	7.52E−01	2.15E+04	2.55E+01	4.77E+00	8.63E+03	5.81E+02	5.95E+02	1.34E−01
1,2,3,7,8,9-HxCDD (pg/g)	1.79E+03	6.37E+02	6.13E+02	2.50E+02	7.08E+02	5.58E+02	8.20E−01	1.23E+04	1.41E+04	7.31E+03	8.95E+04	6.62E+03	4.58E+03	5.35E+01	4.42E+03	1.58E+02	3.79E+01	1.24E+04	5.84E+03	5.95E+02	2.64E+00	7.51E+02	1.28E+01	2.41E+01	1.62E+04	7.77E+02	2.25E+02	7.51E+00	3.18E+03	1.27E+03	2.85E+02	1.73E+04	2.18E+04	1.06E+03	6.07E+00	4.01E+03	7.16E+01	7.76E+00	1.85E+04	1.36E+03	1.40E+03	8.94E−01
1,2,3,7,8,9-HxCDF (pg/g)	3.24E+01	1.32E+01	2.00E+01	2.10E+01	2.45E+01	9.15E+00	< .0491	6.63E+01	4.94E+01	2.21E+01	3.78E+02	1.71E+02	1.41E+02	4.17E−01	1.47E+02	< 2.17	< 1.74	8.89E+02	1.48E+02	< 37	< 0.0481	< 69.7	< 0.355	2.82E−01	5.00E+02	< 43.2	< 34.2	5.16E−01	1.42E+02	7.60E+01	< 41.6	9.44E+02	6.79E+02	7.89E+01	2.61E−01	1.46E+02	< 1.18	8.62E−02	7.27E+02	5.70E+01	4.52E+01	< 0.0514
1,2,3,7,8-PeCDD (pg/g)	1.49E+02	8.37E+01	7.01E+01	4.59E+01	1.46E+02	8.63E+01	1.43E−01	2.33E+02	2.09E+03	1.03E+03	1.81E+03	6.99E+02	5.29E+02	8.47E+00	3.24E+02	1.35E+01	4.08E+00	4.44E+02	7.30E+02	1.27E+02	5.34E−01	6.80E+01	1.59E+00	3.88E+00	1.30E+03	8.28E+01	2.51E+01	1.36E+00	1.53E+02	2.67E+02	< 26.2	3.43E+02	2.41E+03	8.83E+01	6.91E−01	3.19E+02	7.10E+00	1.34E+00	1.08E+03	1.72E+02	1.73E+02	1.74E−01
1,2,3,7,8-PeCDF (pg/g)	4.26E+01	7.24E+01	3.08E+01	3.49E+01	2.60E+02	1.07E+02	1.04E−01	< 20.8	1.31E+02	7.65E+01	3.79E+02	4.31E+02	3.57E+02	7.47E−01	9.95E+02	3.39E+01	4.92E+00	6.08E+02	4.95E+02	< 28.9	1.78E−01	8.36E+01	2.22E+00	1.92E+00	1.29E+03	5.79E+01	3.02E+01	4.21E−01	5.72E+02	2.21E+02	4.57E+01	3.72E+02	2.01E+03	6.72E+01	1.92E−01	1.49E+03	2.65E+00	6.01E−01	1.18E+03	1.21E+02	1.35E+02	< 0.0514
2,3,4,6,7,8-HxCDF (pg/g)	3.73E+02	2.25E+02	1.80E+02	7.18E+01	2.35E+02	1.16E+02	2.04E−01	4.74E+02	1.75E+03	9.12E+02	2.49E+03	1.51E+03	1.13E+03	7.19E+00	8.37E+03	3.35E+02	4.47E+01	2.92E+03	1.71E+03	1.57E+02	3.51E−01	1.55E+03	2.21E+01	1.40E+01	4.17E+03	2.70E+02	5.13E+01	1.48E+00	5.18E+03	3.37E+02	2.86E+02	2.97E+03	5.85E+03	2.51E+02	6.55E−01	1.26E+04	1.84E+01	3.77E+00	5.62E+03	4.15E+02	4.28E+02	9.63E−02
2,3,4,7,8-PeCDF (pg/g)	7.43E+01	2.22E+02	8.36E+01	1.22E+02	9.43E+02	3.86E+02	3.25E−01	< 20.8	2.02E+02	1.09E+02	2.85E+02	4.51E+02	3.72E+02	9.35E−01	7.34E+02	3.04E+01	4.51E+00	3.12E+02	6.76E+02	8.45E+01	1.89E−01	1.08E+02	2.58E+00	2.40E+00	1.19E+03	1.01E+02	3.77E+01	4.69E−01	3.69E+02	2.19E+02	5.33E+01	2.39E+02	1.88E+03	1.27E+02	2.04E−01	1.08E+03	2.15E+00	5.80E−01	8.73E+02	1.43E+02	1.79E+02	8.11E−02
2,3,7,8-TCDD (pg/g)	1.83E+00	1.70E+00	1.47E+00	2.00E+00	6.35E+00	3.73E+00	< .0491	< 15.2	8.63E+01	5.96E+01	7.73E+01	2.33E+01	1.69E+01	5.72E−01	2.65E+00	< .556	1.54E−01	< 19.7	4.59E+01	< 10	7.04E−02	< 20.5	< 0.118	3.18E−01	4.23E+01	< 12.6	< 13.3	2.02E−01	< 19.5	1.93E+01	< 9.53	< 10.6	6.68E+01	< 34.9	< 0.0522	< 24.9	< 0.431	8.96E−02	2.25E+01	< 15.6	< 15.6	5.65E−02
2,3,7,8-TCDF (pg/g)	6.19E+00	1.79E+01	6.16E+00	7.18E+00	6.50E+01	2.76E+01	< .0968	< 75.3	2.28E+01	1.32E+01	5.33E+02	< 85.5	5.540E+01	2.400E−01	9.02E+01	5.43E+00	4.42E−01	< 14.9	8.06E+01	< 16.7	7.60E−02	< 19.4	< 0.405	3.20E−01	2.36E+02	9.77E+00	< 7.67	1.85E−01	4.04E+01	6.04E+01	< 9.39	< 12.3	8.60E+01	< 22.5	6.90E−02	2.11E+02	4.23E−01	< 0.125	< 12.8	< 9.42	2.69E+01	< 0.0514
OCDD (pg/g)	1.13E+06	1.51E+05	1.30E+05	2.51E+04	1.57E+05	NQ	1.45E+02	2.39E+06	1.29E+06	1.05E+06	1.09E+06	1.510E+06	1.010E+06	8.690E+03	1.52E+06	4.12E+04	1.36E+04	1.88E+06	1.57E+06	1.84E+05	3.92E+02	8.06E+05	4.99E+03	2.90E+03	1.84E+06	2.59E+05	6.51E+04	1.04E+03	1.31E+06	3.50E+05	1.10E+05	1.78E+06	1.74E+06	2.96E+05	7.53E+02	1.23E+06	1.80E+04	1.26E+03	1.64E+06	4.10E+05	4.55E+05	1.22E+02
OCDF (pg/g)	1.02E+06	7.70E+04	7.08E+04	2.55E+03	1.54E+04	6.48E+03	1.90E+01	1.16E+06	3.88E+05	1.07E+05	3.71E+05	3.110E+05	2.210E+05	5.700E+02	1.07E+06	7.49E+03	7.29E+03	7.62E+05	2.87E+05	2.48E+04	3.18E+01	5.26E+05	2.11E+03	2.68E+02	6.18E+05	3.93E+04	9.29E+03	9.07E+01	1.44E+06	4.53E+03	1.41E+04	9.18E+05	6.15E+05	4.18E+04	4.66E+01	4.48E+05	4.00E+03	1.17E+02	8.02E+05	6.37E+04	6.98E+04	5.79E+00
TCDD-TEQ (pg/g)*	5.93E+03	1.02E+03	8.41E+02	3.04E+02	1.36E+03	6.40E+02	9.78E−01	1.50E+04	1.24E+04	5.64E+03	3.07E+04	8.395E+03	5.945E+03	4.168E+01	1.67E+04	3.76E+02	9.18E+01	1.85E+04	8.24E+03	8.08E+02	2.33E+00	3.77E+03	3.10E+01	2.39E+01	1.92E+04	1.12E+03	3.01E+02	6.36E+00	1.22E+04	1.62E+03	5.38E+02	2.06E+04	2.56E+04	1.31E+03	3.72E+00	1.43E+04	7.20E+01	7.79E+00	2.52E+04	1.80E+03	2.02E+03	7.46E−01
Pentachlorophenol (mg/kg)	1.90E+03	3.50E+01	3.40E+01	2.30E+00	8.60E+00	3.80E+00	1.50E−01	8.10E+03	3.20E+02	3.50E+01	3.10E+02	5.000E+01	4.700E+01	< 0.580	5.80E+02	4.70E+00	1.90E+00	2.40E+03	1.70E+02	6.30E+00	< 0.240	2.90E+02	4.30E−01	1.90E−01	8.20E+02	1.60E+01	3.60E+00	< 0.360	1.50E+03	3.50E+01	4.30E+00	2.40E+03	1.20E+03	2.70E+01	< 0.460	4.90E+02	5.00E−01	< 0.180	2.90E+03	4.90E+01	5.10E+01	< 0.0660

*TCDD-TEQs are based on human health using Toxic Equivalency Factors from van den Berg et al. (2006)

**Table 2:** Arithmetic mean (standard deviation) concentration of pentachlorophenol and 2,3,7,8-tetrachlorodibenzo-*p*-dioxin equivalents in surface soil of Kenai National Wildlife Refuge and various regulatory values for comparison

		EPA Eco	ADEC HH^a^	ADEC Mig GW^b^
Pentachlorophenol (ppm dry wt)				
At pole	1810 (2210)	2.1 (USEPA 2007)	13	0.0043
25 cm	157 (341)			
50 cm	17.8 (19.5)			
TCDD-eqs-HH^c^ (ppt dry wt)				
At pole	15,200 (8790)	3.15 (USEPA 2018)	60	3.9
25 cm	5170 (7610)			
50 cm	1510 (2080)			
Background	9.31 (16.0)			

^a^Alaska Department of Environmental Conservation Statute 18 AAC 75.340: Method Two clean-up levels for soil based on human health (direct contact)

^b^Alaska Department of Environmental Conservation Statute 18 AAC 75.340: Method Three clean-up levels for soil based on human health—migration to groundwater

^c^2,3,7,8-TCDD equivalents based on human health risk, using WHO toxic equivalence factors (van den Berg et al. 2006)

**Fig. 2:**
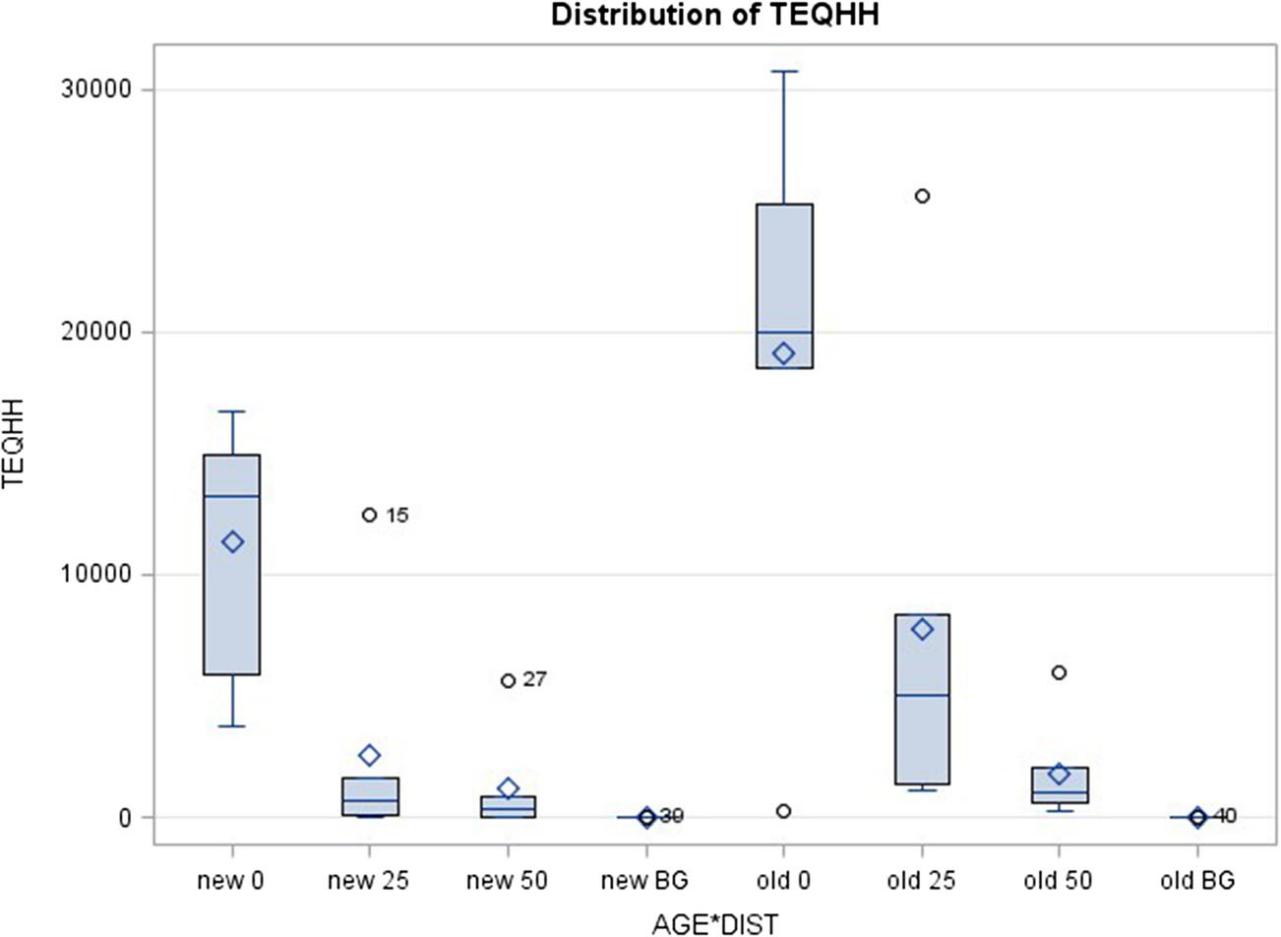
Distribution of TCDD-TEQ levels in surface soils (ppt d.w.) at three lateral distances (0, 25, 50 cm) from old (> 50 years) and new (< 25 years) pentachlorophenol-treated utility poles with accompanying soil background levels (SAS GLM output). Upper and lower bounds of the shaded box represent the sample 75th and 25th percentiles; line within box is the sample median, and diamond is the sample mean. Whiskers outside box represent range of data within 1.5 inter-quartiles; data outside this range are represented by circles, with adjacent number indicating data ID

The ANOVA (*F* = 8.77, *df* = 7,40, *P* < 0.001) revealed that TCDD-TEQs within the surface soil samples varied by the age of the poles (*P* = 0.043) and distance from those poles (*P* < 0.001) but not their interaction (*P* = 0.285) (Fig. 2). Mean surface soil TCDD-TEQ levels adjacent to “old” (> 50 years) poles were nearly twice as high (mean = 7180 ppt, SD = 10,100) as levels adjacent to “new” (< 25 years) poles (mean = 3780 ppt, SD = 5730). TCDD-TEQs decreased with distance from the pole, averaging 15,200 ppt (SD = 8790) at the pole base, 5170 ppt (SD = 7610) at 25 cm from poles, and 1510 ppt (SD = 2080) at 50 cm from poles. The mean TCDD-TEQ level in background samples was 9.3 ppt (SD = 15). Surface soil levels of pentachlorophenol and TCDD-TEQs were above Alaska Department of Environmental Conservation (ADEC) Method Two soil clean-up levels for protection of human health at all distances sampled (Table 2). Mean surface soil levels of TCDD-TEQs exceeded USEPA ecological screening levels at all distances sampled (Table 2).

Total organic carbon in soil samples ranged from 0.98 to 54.4% (Table 1). The characteristics of the Kenai soils, as noted in field observations and total organic carbon content, led us to classify the predominant soil type as organic rather than sand or clay.

Laboratory performance was acceptable for the blind duplicate soil samples. The relative percent difference for the two pentachlorophenol blind duplicate pairs was 39 and 15%. The relative percent difference for the two TCDD-TEQ blind duplicate pairs was 14 and 10%.

## Discussion

Prior to undertaking this project, we had two competing and opposing hypotheses regarding whether soil surrounding pentachlorophenol-treated poles installed in the 1950s would be more or less contaminated than soil surrounding poles installed less than 20 years ago. We hypothesized that soil might be more contaminated with dioxins and furans in soils surrounding the poles from the 1950s, because pentachlorophenol mixtures manufactured prior to 1987 were known to contain higher concentrations of dioxins and furans than newer products do. Alternatively, we hypothesized that soils surrounding the poles from the 1950s might be less contaminated than soils surrounding newer poles, because contaminants from the older poles have had so much longer to weather and degrade in the environment. Our results showed that despite the passage of over 50 years since pole installation, TCDD-TEQs were still present in surface soils near old poles, at levels greater than those found near newer poles. This finding points both to the remarkable environmental persistence of PCDDs and PCDFs in Kenai soils and to the relative severity of dioxin/furan contamination of pentachlorophenol mixtures in wood treatment products from the late 1950s/early 1960s.

Although pentachlorophenol and TCDD-TEQ levels in surface soils decreased significantly with distance from the poles in this project, levels of both contaminants exceeded State of Alaska clean-up levels for all poles even at the farthest distance sampled (50 cm). The nature and extent of soil contamination was not fully characterized during this project, because soils were not sampled at depth or at a great enough distance to delineate the complete lateral extent of contamination. Thus, additional sampling is warranted, both at depth and at greater distances from the pole, to characterize the full scope of soil contamination around the poles.

It is unknown whether the poles we sampled are representative of pentachlorophenol-treated utility poles located elsewhere. The pentachlorophenol and TCDD-TEQ levels we detected in surface soil near new utility poles on KENWR were similar to, but lower than, levels found in surface organic soils in the Montreal Quebec area near poles less than 20 years old (Bulle et al. 2010). Montreal’s latitude is 15° further south than KENWR. Pentachlorophenol, dioxins, and furans might be more persistent in the cold soils of the Kenai Peninsula relative to the warmer soils found in many parts of the USA. However, our study and that of Bulle et al. (2010) provide cause for concern, because they demonstrate the possibility that many of the millions of utility poles in North America may each be point sources of pentachlorophenol and dioxin/furan soil contamination. This may cause a problem both in terms of potentially unacceptable risk, and from the perspective that dioxin-contaminated soil is costly to remediate.

In 2009, the Vermont Department of Health responded to two separate incidents of private drinking water contamination with pentachlorophenol from treated utility poles (Karlsson et al. 2013). In both cases, utility poles upgradient from the drinking water source had been recently replaced, and an odor in their water alerted residents to the presence of a contaminant. In one residence with a shallow well, the water had a level of 2.06 mg/L of pentachlorophenol, which was about 2000 times greater than the EPA maximum contaminant level of 0.001 mg/L. In the second household in a different area, which obtained its drinking water from a private spring, a pentachlorophenol level of 0.007 mg/L was documented from the tap. The Vermont Department of Health did not analyze the drinking water from either household for potential contamination with PCDDs or PCDFs. Nevertheless, their work documented that drinking water contamination can occur from pentachlorophenol-treated utility poles, at levels that may pose a risk to human health.

In May 2015, at the seventh meeting of the Conference of the Parties to the Stockholm Convention on Persistent Organic Pollutants (POPs) in Geneva, Switzerland, a final decision (UNEP/POPS/COP.7-SC-7/13) was made to list pentachlorophenol and its salts and esters in Annex A, with specific exemptions for the production and use of pentachlorophenol for utility poles and cross arms. The Stockholm Convention calls for international action to eliminate or restrict the production or use of specific listed POPs, and decisions are binding on the 179 signatory countries. An Annex A listing is the most restrictive category of the Convention, calling for the elimination of the production and use of listed POPs. The United States has not ratified the Convention, and is not bound by the Convention’s decisions. The U.S. Environmental Protection Agency (EPA) currently allows the use of pentachlorophenol-treated wood for utility poles; the re-registration of pentachlorophenol as a pesticide for this use is reviewed periodically. The U.S. EPA last renewed the registration of pentachlorophenol for wooden poles and cross arms under the Federal Insecticide, Fungicide and Rodenticide Act in 2008 (USEPA 2008).

While pentachlorophenol-treated utility poles pose a degree of risk to human health and the environment, they also provide effective infrastructure for the delivery of electricity throughout North America. The decision to continue to install pentachlorophenol-treated poles requires an analysis of alternatives, relative risks, and cost. Other wood preservation chemicals are available for use, but often pose their own health and environmental risks. For example, copper, arsenic, and other inorganic chemicals are common ingredients in wood preservation products such as copper naphthenate, ammoniacal copper zinc arsenate, ammoniacal copper arsenate, chromated copper arsenate, and ammoniacal copper quaternary (Hutton and Samis 2000). Copper-containing utility poles may be unacceptable for use in wetland environments that sustain early life stages of fish, such as in the Kenai NWR ROW, because copper is toxic to fish at very low concentrations. There are also alternatives to the use of treated wood for utility poles, such as non-treated cedar poles, cement, fiberglass, spun concrete, metal, or buried wires.

Site-specific environmental characteristics must be factored in to select the most appropriate material for a particular project. Life-cycle assessment can be a useful tool to compare the environmental impacts of various pole alternatives from “cradle to grave,” including the growth or manufacture of the pole, transportation, time in use, and disposal following decommissioning. Many factors can be considered, including greenhouse gas emissions, fossil fuel use, acidification, water use, eutrophication, ecological toxicity, etc. A recent life-cycle assessment compared pentachlorophenol-treated wooden utility poles with steel and concrete utility poles (Bolin and Smith 2011). While it found that pentachlorophenol-treated poles compared favorably in several respects, it unfortunately did not include consideration of dioxin and furan impurities, their potential impacts on the environment, or the potential cost of contaminant remediation. Similarly, another life-cycle assessment that compared steel and concrete utility poles with Veneer-based composite (VBC) poles did not consider the potential environmental toxicity associated with the preservative used in the VBC poles (alkaline copper quaternary) (Lu and El Hanandeh 2017). Consideration of environmental toxicity impacts would be a valuable addition to future life-cycle assessments examining the environmental impacts of utility poles.

## Conclusions and recommendations

Utility poles are present in many environments with potential human receptors, including parks, schools, playgrounds, and backyards. Vulnerable human receptors in these environments may be being exposed to unacceptable levels of pentachlorophenol and dioxin/furans, from touching contaminated poles, exposure to contaminated soils, or from consumption of contaminated drinking water. Additional research is needed to characterize soil contamination surrounding utility poles in other habitat types throughout the USA and determine whether soils in the continental USA are similarly contaminated. Further characterization of the risks posed to human health and the environment should also be undertaken.
